# Public Hearing Aid Policies in Brazil: Operational Gaps, Replacement Costs, and Data Governance in the Unified Health System (SUS)

**DOI:** 10.1055/a-2858-5997

**Published:** 2026-09-01

**Authors:** Ricardo Ferreira Bento, Mara Edwirges Rocha Gândara, Silvio Pires Penteado

**Affiliations:** 1 Department of Otorhinolaryngology, School of Medicine, Universidade de São Paulo, São Paulo, SP, Brazil

**Keywords:** hearing rehabilitation, public health practice, hearing aids, health care rationing, conflict of interest

## Abstract

**Introduction:**

The Brazilian Unified Health System (SUS, from the Portuguese Sistema Único de Saúde) provides free hearing aids (HAs), including otological evaluation, audiological therapy, audiological exams, and ear molds through authorized centers. In addition to the complexity and high investment costs, there is no centralized government agency to manage these resources, so each authorized center works on its own. On the other hand, HAs providers are independent distributors or subsidiaries driven by profit and key-performance indicators in their best interest.

**Objectives:**

To analyze the operational efficiency of SUS in the provision of HAs and explore potential improvements through digital data governance and optimization of replacement rates through simulations.

**Data Synthesis:**

We accessed national data from 2008 to 2024 through the Ministry of Development, Industry and Foreign Trade (COMEX/MDIC) and SUS's Department of Informatics (DATASUS). We integrated these sources with operational data from Reouvir, a leading SUS-accredited center, to model cost scenarios and identify management inefficiencies. Cases from SUS accounted for an average of 49.9% of Brazil's HA imports (3.48 million units, US$437 million). However, high replacement rates—some exceeding 70%—suggest inefficiencies. Simulations indicate that reducing replacement rates by 5 to 20% could yield annual savings of US$1.23 to 5.15 million or enable an increase of 9,780 to 41,026 new fittings per year, respectively.

**Conclusion:**

Despite its robust public policy, SUS lacks performance monitoring to enable suppliers to shape market dynamics. Improved digital governance could enhance cost-efficiency and access.

## Introduction

Over 1.5 billion people globally experience hearing loss, with projections estimating 2.5 billion by 2050 and annual productivity losses reaching US$1 trillion. 1 In Brazil, disabling hearing loss is estimated at 5.2%, affecting over 11 million individuals. 2

Bento et al. 3 express hearing loss is linked to a range of etiologies—prenatal (e.g., rubella), perinatal (e.g., anoxia), and postnatal (e.g., meningitis)—with strong associations with cognitive decline. 4 5 Despite hearing aids (HAs) being a cost-effective solution, 6 7 their uptake remains low due to costs, 8 9 stigma, 10 and system-level barriers, 11 batteries, 12 and failure to attend previously scheduled appointments. 13

The Brazilian public health system (Sistema Único de Saúde, SUS, in Portuguese) offers free HAs and audiological services through accredited centers. 14 Devices are categorized by technology level as A (lower-end), B (mid-range), and C (high-end) and procured from major international suppliers. The fixed pricing model, unchanged since 2004, may limit innovation and efficiency. Most devices are imported and regulated via the NCM code 90214000.

In Brazilian law, Administrative Rule 589 (October 8, 2004) in paragraph § 4 stipulates replacements of HAs will be authorized only in cases of theft, technical failure, and proven progressive hearing loss. Theft must be proven by presenting a police report; technical failure must be documented by the company's repair estimate; and progressive hearing loss must be proved by a medical report.

## Review

### Data Collection

National data were retrieved from the Ministry of Development, Industry and Foreign Trade (COMEX/MDIC), with the imported data being filtered by NCM code 90214000, excluding cochlear implants and bone conduction devices. As well as SUS's Department of Informatics (DATASUS) system Tabnet, with HA-related procedures (excluding accessories and nonelectronic devices) from 2008 to 2024. Finally, Reouvir's (Hospital das Clínicas, FMUSP) internal data, including brand-level usage, replacement rates, and patient follow-up records, were also compiled.

The present study was approved by the university's Ethics Committee under protocol 6.390.430, October 5, 2023.

### Data Processing

Data were harmonized into Microsoft Excel (Microsoft Corp.) databases, with Supplementary Material Appendix A presenting yearly total imports versus SUS acquisitions by state; and Supplementary Material Appendix B reporting replacement scenarios using Reouvir's average 32% rate, simulating 5 to 20% reductions and associated cost and access gains.

### Simulations

Cost savings and increased HA availability were modeled using a fixed reimbursement value (US$125.45 for B-level technology) and an exchange rate of R$5.28 to US$1.

### SUS Participation in the National HA Market

**Fig. 1:**
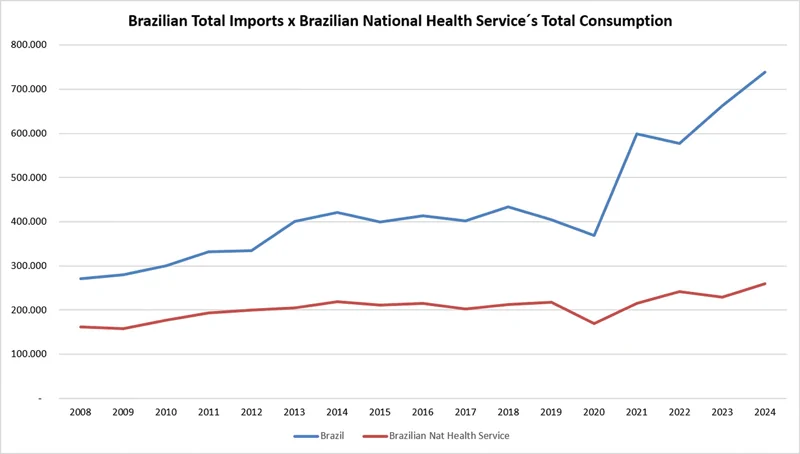
Brazilian imports versus SUS consumption (2008–2024).

From 2008 to 2024, Brazil's HA imports rose 172.1%, from 271,429 to 738,730 units. In SUS, the procurement increased 59.9% in the same period (162,130–259,268), with its market share dropping from 59.7 to 35.1% ( Fig. 1 ).

The COVID-19 pandemic caused a 22.3% drop in SUS acquisitions in 2020. However, the following year saw partial recovery.

### State-level Disparities

**Fig. 2:**
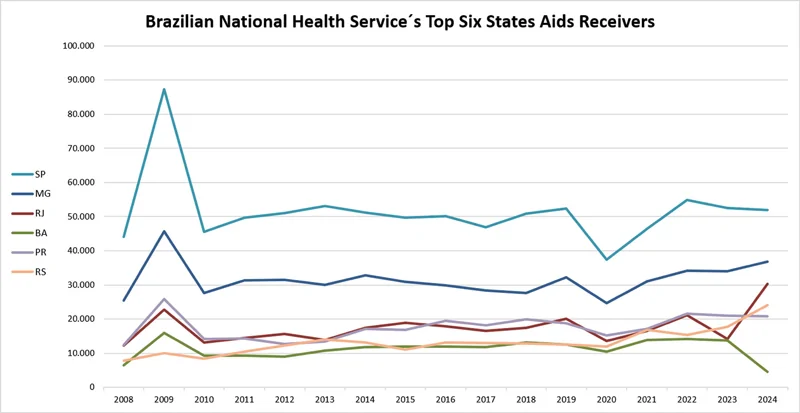
Brazilian Public Health Service's top six hearing aids receivers, per state.

States like São Paulo and Minas Gerais dominate HAs consumption, while northern states like Roraima and Amapá received negligible volumes. In 2024, São Paulo alone accounted for 25.1% of SUS distribution ( Fig. 2 ).

### Replacement Rate Simulations

We built a spreadsheet containing total import data and national consumption figures for SUS in the period between 2008 and 2024 ( Supplementary Material Appendix B ). The second column shows Brazil's total imports, and the third shows SUS's total consumption for the same period. In the 4 ^th^ column, the number of HAs allocated to new patients was labeled as “New Cases”, this time based on Reouvir's 32% replacement rate.

For example, in the first row, SUS reports 162,130 new HAs (3 ^rd^ column), but with a 32% replacement rate this number would drop to 110,248 HAs (4 ^th^ column). The resulting difference of 51,822 HAs is presented at the 5 ^th^ column. The same logic applies to the following years. As shown in the Supplementary Material Appendix B , the cells in blue cover this rationale.

Hypothetically, the results of a 5% reduction in the replacement rate from the baseline of 32%, which would result in a new rate of 27% and a subsequent 73% increase in “New Cases”. In the 6 ^th^ column, 2 ^nd^ row, we have 118,355 new cases, from 162,130 (3 ^rd^ column) multiplied by 0.73 (73%). This time, the new replacement will set as 43,775 HAs, a result from 162,130 HAs (third column) multiplied by 27%. “Increased New Cases” will bring the total HAs acquired with 5% efficiency increase. Thus, 8,107 is the difference between this new figure of 118,355 and 110,248 (the 6 ^th^ column minus the 4 ^th^ column, same row). The same logic applies to the following years. But now we have a new column showing the financial gain resulting from a 5% increase in efficiency (9 ^th^ column). Simply multiply the increase in new HAs (8,107) by the average amount of R$ 700 that the government pays for B-level technology HAs, resulting in savings of R$ 5,674,550 for that year. In Supplementary Material Appendix B , the cells in green cover this rationale. The same logic applies to the efficiency rates of 7.5 (shown in salmon), 12 (brown), and 20% (gray).

Simulated scenarios showed in a year-basis considerations: 5% reduction: US$1,26 million savings or +9,780 new HAs; 7.5% reduction: US$ 1,93 million/year savings or +15,380 HAs; 12% reduction: US$ 3.09 million/year savings or +24,600 HAs; and, finally, 20% reduction: US$ 5.15 million/year savings or +41,000 HAs.

Therefore, a 5% reduction meant more HAs than the state of Tocantins received during the entire period discussed. Also, a 7.5% reduction accounts for 89.9% of all HAs acquired by SUS in Brazil's Midwest region in 2024 (MS, MT, GO and DF). The 12% reduction exceeds the consumption of the 5th-largest state in terms of SUS usage in 2024, Rio Grande do Sul (RS). Finally, a 20% reduction means 79% the consumption of the largest state in terms of SUS usage in 2024, São Paulo (SP).

## Discussion

There is a strong social commitment in SUS to ensure cost-efficiency and oversight, though it lacks the necessary mechanisms. Distributors capitalize on this structural gap, resulting in high replacement rates and market domination by a few suppliers. Authors are critical of the concentration of HAs production in the possession of a very small number of companies, 15 16 notably through control of supply and demand. 17

The fixed reimbursement model and outdated technology classification limit innovation. Furthermore, the frequent discontinuation of fitting software by vendors makes reprogramming difficult, raising questions about whether such replacements can be considered reasonable, since they are not listed in Administrative Rules 587 and 589 (October 2004).

Reouvir clinic recently developed a set of key-performance indicators (KPIs) to better understand replacements, such as the number of replacements by brand and model, cost estimates, requests for replacements instead of repairs, and the lifespan of HAs by brand and model, among other metrics. At the time of this publication, however, we have not yet been able to compile a sufficiently large dataset to support a conclusion.

Routinely, Reouvir has implemented patient interviews and cost-sharing inquiries to curb unnecessary replacements, yet 95% of users report inability or unwillingness to pay for repairs.

The present study has some limitations. Our findings are based on Reouvir's experience, which, despite its size and history, may not reflect national heterogeneity. Wider data sharing across centers is essential for a comprehensive assessment.

## Final Comments

While SUS operates a large-scale HA adaptation program, it still lacks the mechanisms to ensure cost-effectiveness and standardization. Optimizing replacement rates could substantially increase access and reduce public expenditure.

We recommend creating a national dashboard ranking centers by efficiency indicators, regular price audits to realign reimbursement rates, and open-format HA programming software, as well as defining supplier obligations.
